# Mixture Analysis at Micromolar Concentrations with 2D Benchtop NMR Enhanced by SABRE Hyperpolarization

**DOI:** 10.1021/acs.analchem.6c02720

**Published:** 2026-08-13

**Authors:** Bono O. Jimmink, Mattia Negroni, Thom B. Posthumus, Arno P.M. Kentgens, Marco Tessari

**Affiliations:** Institute for Molecules and Materials (IMM), 6029 Radboud University, Heyendaalseweg 135, Nijmegen 6525 AJ, The Netherlands

## Abstract

Because of its low cost and portability, benchtop NMR has become increasingly popular over the last years. However, these low-field instruments lack sensitivity and spectral resolution, which limits their application in real-world problems, particularly at low concentrations. Here, we combine SABRE hyperpolarization with two-dimensional NMR spectroscopy on a benchtop spectrometer to gain sensitivity and spectral resolution. We employ a novel cosubstrate approach, utilizing an α-amino acid, which facilitates measurements at low micromolar concentrations. This approach enabled the complete resolution of a mixture of six N-heteroaromatic compounds at concentrations of 5 μM each, within 20 min. The power of this methodology was further tested on an extract of ground coffee, for which several analytes – estimated in the low micromolar concentration range – were identified.

## Graphical abstract

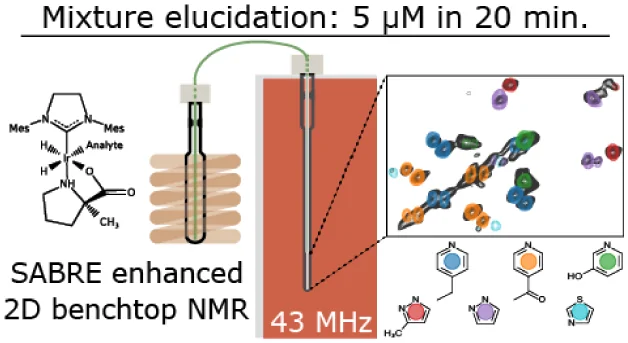

## Introduction

Two-dimensional experiments have long been used to enhance the resolution and facilitate structure identification in NMR spectroscopy.^1^ Resolving power becomes increasingly important for low-field (43–100 MHz^1^H resonance frequency) “benchtop” NMR spectrometers, which offer reduced chemical shift dispersion compared to their high-field counterparts.^2^ While there has been recent interest in the use of benchtop spectrometers for the analysis of complex mixtures and metabolomics,^2–4^ 2D NMR experiments have been scarcely employed for such cases. Notable uses of 2D NMR for mixture analysis using benchtop spectrometers include the ultrafast COSY experiments on vegetable oils by Giraudeau and coworkers,^5^ the 2D *J*-resolved experiment on groundwater samples by Fallaise et al.^6^ and the (conventional) COSY experiment on human urine by Leenders et al.^7^

One reason for the underutilization of 2D benchtop NMR for chemical analysis is its limited sensitivity, generally restricting its application to compounds in the millimolar range.^8,9^ Recently, methods based on Parahydrogen Induced Polarization (PHIP) have been applied to address this sensitivity limitation,^10–16^ showing promise for the analysis and quantification of mixtures.^17,18^ Signal Amplification by Reversible Exchange (SABRE) is one such PHIP-based technique, offering the key advantage of repeatable hyperpolarization thereby enabling multiscan acquisition.^19,20^ SABRE enhancement of proton magnetization occurs spontaneously at fields of approximately 6 mT, as *para*-enriched hydrogen and analyte molecules reversibly associate with an iridium complex, creating a transient scalar coupled network.^21^ Once the analyte dissociates, it can be detected by NMR spectroscopy with greatly increased signal intensity. SABRE has been shown to work for a range of analytes and is particularly effective for N-heteroaromatic compounds.^22^ A decade ago, Eshuis et al. introduced a strategy for the NMR measurement and quantification of dilute analytes via SABRE through the addition of a large excess of coligand to the solution.^23^ This so-called “cosubstrate” prevents the formation of SABRE inactive complexes under dilute analyte conditions, producing a linear dependence of NMR signals on analyte concentration, enabling quantification through standard addition.^23,24^ Previous publications within the field of SABRE-benchtop NMR have focused on the analysis of dilute mixtures by one-dimensional NMR experiments,^11,17,18,25^ while two-dimensional experiments have predominantly been performed on pure compounds at relatively high concentrations.^26,27^

Here, we extend SABRE hyperpolarization to 2D benchtop NMR for the analysis of mixtures of N-heteroaromatic compounds at micromolar concentration. To this end, we chose the 2D TOCSY (total correlation spectroscopy) experiment because of its general applicability. 2D TOCSY experiments are widely used at high field, where they produce well-defined patterns that readily reflect the protons topology, providing detailed structural information for analyte identification.^28,29^ At low field, not surprisingly, the reduced chemical shift dispersion in combination with the associated strong coupling regime result often in complex and nonintuitive 2D TOCSY patterns which can seriously hamper spectral analysis. This issue becomes even more significant when dealing with mixtures, making spectral databases crucially important for analyte identification at low field. Nevertheless, 2D SABRE-TOCSY provides a far superior signal dispersion than 1D spectra for benchtop NMR, as demonstrated here for an artificial mixture of aromatic compounds at low micromolar concentration.

This approach was further tested on a complex mixture, namely a methanolic extract of ground coffee. During the roasting of coffee beans, a multitude of SABRE-amenable compounds – such as pyrroles, pyrazines, thiazoles and pyridines – are formed.^30^ We show how several such compounds – estimated at low micromolar concentrations – can be identified from the hyperpolarized 2D TOCSY experiment. This constitutes the first application of SABRE-enhanced 2D NMR for the structural elucidation of analytes in a complex mixture.

Our results clearly indicate that benchtop 2D NMR, in combination with SABRE hyperpolarization, is a viable route to increase resolving power, with a limit of detection approaching nanomolar concentrations. These advances bring applications such as complex mixture analysis within reach of benchtop spectrometers, although – as will be outlined below – several limitations still need to be overcome.

## Experimental Section

### SABRE Hyperpolarization

Hyperpolarization was performed using an automated pneumatic transfer system, as detailed in previous work.^17^ Two vessels – one at 7 mT and one inside the benchtop spectrometer – are kept at 5 bar hydrogen pressure. An Arduino MEGA 2560 controls a series of solenoid valves which regulate sample transfer by inducing a pressure difference between the two vessels.

The SABRE procedure is as follows: (I) Samples are deoxygenated by bubbling nitrogen gas through the solution for 5 min. (II) The catalyst precursor complex is activated by bubbling *para*-enriched hydrogen gas through the solution for 5 min. (III) The sample is heated to 50 °C for 10 min using a water bath to accelerate the formation of the axial/equatorial bidentate association of the amino acid to the iridium catalyst *(see below)*. (IV) Samples are then cooled to room temperature and are thereafter ready for measurement.

SABRE experiments are performed by bubbling 51% *para*-enriched hydrogen gas through the solution for 4 s at a field of 7 mT. Thereafter, the sample is transferred to the observation field in approximately 3 s. After 1 s to dampen convection motions in the sample, the spectrometer is triggered for measurement.

### NMR Samples

SABRE samples of analytes at low-micromolar concentration contained 100 μM [Ir­(SIMes)­(COD)]Cl catalyst precursor, 250 μM l-proline as cosubstrate as well as 10 mM piperidine (pH 11.07) buffer in CD_3_OD.

Samples for individual analyte measurements at thermal equilibrium contained 10 mM or 50 mM of pure analyte in CD_3_OD.

The SABRE sample for coffee extract contained 450 μM [Ir­(SIMes)­(COD)]­Cl catalyst precursor, 1125 μM l-proline as cosubstrate, 10 mM piperidine buffer (pH 11.07) in CD_3_OD. 600 μL methanolic coffee extract (see Supporting Information) was used for the sample with a total volume of 1500 μL, meaning a dilution factor of 2.5× for the coffee extract.

All NMR samples were spiked to 0.5% protonated methanol to facilitate shimming, using the Magritek “quickshim” procedure.

### Benchtop NMR Spectroscopy

All benchtop experiments were performed at room temperature on a Magritek Spinsolve spectrometer operating at 43 MHz ^1^H resonance frequency. One-dimensional SABRE spectra were acquired by accumulating 8 transients in ∼2 min. Data processing was performed using ssNake,^31^ and consisted of Lorentz-to-Gauss transformation and zero-filling to 65k points prior to Fourier Transform and polynomial baseline correction.

For the 2D TOCSY data sets, phase modulation of the signal was achieved via the Preservation of Equivalent Pathways (PEP) scheme, as originally introduced by Cavanagh and Rance.^32^ While directly providing sign discrimination in the indirect dimension, this approach suffers from undesirable phase-twisted lineshapes that negatively affect the spectral resolution. This issue is alleviated by application of a pseudoecho transformation (e.g., a sin^2^ apodization function) to produce an envelope that is symmetrical about the center of both time dimensions. As a result, no dispersive component is obtained after Fourier transformation, which results in a higher resolution of the absolute-value spectrum.^33,34^

Imbalance between the two orthogonal components in the PEP scheme produces quadrature artifacts, which can become noticeable in the presence of large signals, as observed for the sample of coffee extract. In this case we opted for achieving sign discrimination in the indirect dimension via TPPI to avoid such artifacts altogether.

A DIPSI-2 ^35^ period of 77 ms with a bandwidth of 15 kHz was used for isotropic mixing in the 2D TOCSY experiment. The 2D data sets were acquired with one scan per increment and consisted of 80 (t_1_, real) × 4096 (t_2_, complex) and 256 (t_1_, real) × 4096 (t_2_, complex) points for the spectra shown in Figures 4 and 5, respectively. Data processing was performed using nmrPipe^36^ with sin^2^ apodization in both dimensions, zero-filling to 1024 (t_1_, real) × 32768 (t_2_, complex) and Fourier Transform. Spectra are reported in power mode (S = real^2^ + imaginary^2^). In the case of Figure 5, a low pass solvent filter was used to remove signals originating from methanol and dissolved hydrogen, and the NEAS algorithm was applied to reduce t_1_-noise.^37^

### Theory

When working with dilute solutions, an excess of coligand (referred to as cosubstrate) is usually necessary to ensure the formation of SABRE-efficient catalyst-analyte complexes, as previously detailed.^20^ While this approach can provide SABRE spectra at submicromolar concentrations, the large excess of cosubstrate determines a competition with the analytes for catalyst association that negatively impacts on the signal enhancement.

In the present work, α-amino acids have been employed for the first time as cosubstrates for SABRE hyperpolarization. The association of α-amino acids with an iridium complex in combination with heteroaromatic substrates has been described in detail using both NMR and DFT methods, with the so-called “bidentate axial/equatorial” binding being the thermodynamically favored form (see Figure 1).^38–40^

**Figure 1:**
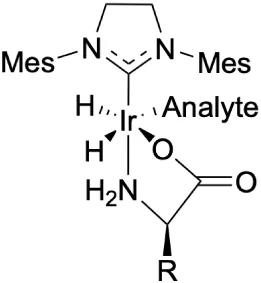
Sketch of the SABRE-active complex [Ir­(SIMes)­(α-amino acid)­(analyte)­(H)_2_]. The thermodynamically favored bidentate axial/equatorial binding mode is shown for the α-amino acid. The *Analyte* ligand refers to a heteroaromatic compound.

Due to this chelating mode of association, α-amino acids display a much higher affinity for the catalyst and can, therefore, be employed at substantially lower concentration excess than conventionally employed cosubstrates. Consequently, the nonoccupied equatorial site (see Figure 1) is available for analyte association, with negligible competition from the cosubstrate. This represents an important advantage with respect to previously employed cosubstrates, e.g., 1-methyl-1,2,3-triazole (mtz),^23^ DMSO^41^ or perdeuterated pyridine.^26^ Interestingly, the chelating ligand acethydrazide has recently been proposed as cosubstrate for improved SABRE enhancements, allowing for submicromolar detection.^42^ Based on the comparison of DFT structures with experimental data, the authors proposed a different chelating mode than that of amino acids, which is nevertheless consistent with the reduced competition with respect to traditional cosubstrates, as given above.

Furthermore, as the equatorially bound carboxyl moiety does not contain protons, no spin dilution occurs during SABRE, maximizing the analytes hyperpolarization.^43–45^ Rather than utilizing [Ir­(IMes)­(COD)]­Cl as catalyst precursor, we opted for [Ir­(SIMes)­(COD)]Cl to reduce *ortho*-directed hydrogen isotope exchange in the course of the experiments.^46–48^ As previously demonstrated, this process is negligible when using [Ir­(SIMes)­(COD)]Cl as a precursor, compared to the more widely used [Ir­(IMes)­(COD)].^17,46^

## Results and Discussion

Figure 2 (orange) shows the SABRE spectrum of six compounds, each at 50 μM in deuterated methanol acquired in ∼2 min in the presence of l-proline as coligand, displaying NMR signals enhanced ca. 2000 times with respect to thermal equilibrium. For the sake of comparison, Figure 2 also shows the SABRE spectra of the same mixture using pyridine-*d*_5_ (10× excess with respect to catalyst, dark blue) or DMSO-*d*_6_ (5× excess with respect to catalyst, turquoise) as cosubstrate, as reported in recent publications focusing on trace analysis.^11,17^ As seen in the overlay of the spectra, using l-proline as a coligand for SABRE hyperpolarization results in a significantly increased overall signal intensity, outperforming the other two strategies.

**Figure 2:**
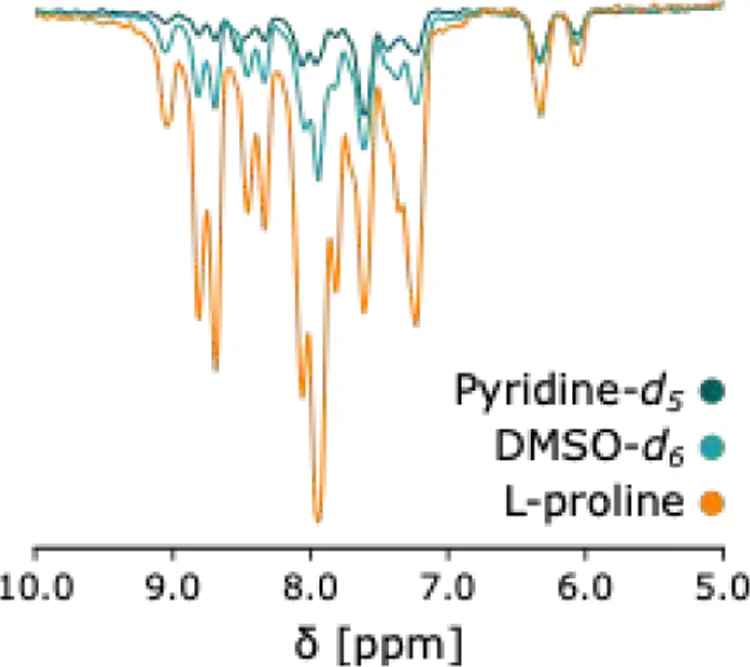
Overlay of SABRE spectra of a mixture of 4-acetylpyridine, thiazole, 3-hydroxypyridine, 4-ethylpyridine, 3-methylpyrazole and pyrazole, each at 50 μM, hyperpolarized using 100 μM catalyst. The spectra were acquired using 200 μM l-proline as coligand (orange), or 1000 μM pyridine-*d*_5_ (dark blue), or 500 μM DMSO-*d*_6_ (turquoise) as cosubstrate.

A similar enhancement was observed by exchanging l-proline for glycine, suggesting this high SABRE efficiency to be a general property of complexes of the type [Ir­(SIMes)­(α-amino acid)­(analyte)­(H)_2_] (see Supporting Information).

SABRE ^1^H hyperpolarization is generally performed at a polarization field of 6 mT, based on the avoided level crossing (LAC) condition of complexes of the type [Ir­(IMes)­(pyridine)_3_(H)_2_].^49,50^ To determine the optimal SABRE conditions for [Ir­(SIMes)­(α-amino acid)­(analyte)­(H)_2_] complexes, a mixture of 3-hydroxypyridine and pyrazine were hyperpolarized at various polarization fields. The signal integral of both analytes as a function of polarization field is shown in Figure 3. The optimal polarization field for both analytes align very well and is situated at approximately 7 mT. This increase in the avoided level crossing condition is consistent with the larger interhydride couplings (∼9 Hz)^38^ present in complexes such as shown in Figure 1.^51^

**Figure 3:**
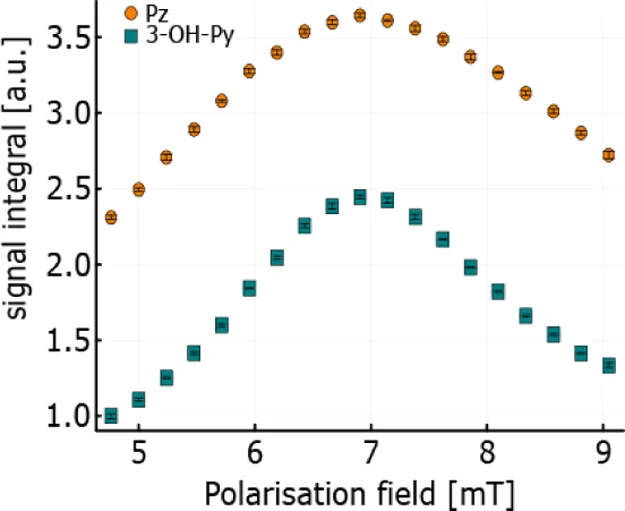
Signal integral of 3-hydroxypyridine (3-OH-Py, blue) and pyrazine (Pz, orange) as a function of polarization field. The data was normalized such that the first point of the 3-hydroxypyridine data set corresponds to a value of 1.0. Both analytes were 75 μM and hyperpolarized using 100 μM SIMes catalyst, 250 μM l-proline and 10 mM piperidine buffer (pH 11.07). Data points represent the average signal integral of 5 scans, with error bars representing the standard deviation.

While the sensitivity issue can be addressed – at least for certain classes of analytes – by SABRE hyperpolarization, signal overlap remains a major limitation in benchtop NMR.

This can be reduced by combining SABRE hyperpolarization with two-dimensional NMR spectroscopy. As previously demonstrated, spin repolarization can be achieved at the beginning of each transient by automatic pneumatic shuttling of the sample between a vessel at 7 mT – where *para*-enriched hydrogen is bubbled through the solution for a few seconds – and the benchtop spectrometer.^17^ This allows for signal averaging as well as the acquisition of multidimensional NMR spectra.

In the present case, the 2D TOCSY experiment was chosen for its general applicability, structural information, high sensitivity and straightforward implementation. The sensitivity and resolution can be appreciated in the spectrum shown in Figure 4 (black contours), which was acquired in ca. 20 min on a mixture of six heteroaromatic analytes, at a concentration of 5 μM each. Even at such low concentrations, SABRE results in signals well above the noise level, with S/N ratio’s ranging between 50–300. Such sensitivity for proton-detected SABRE has previously only been reported for experiments performed at high magnetic field strengths,^23,42^ where the S/N ratio benefits from detection at higher frequencies.^52^

**Figure 4:**
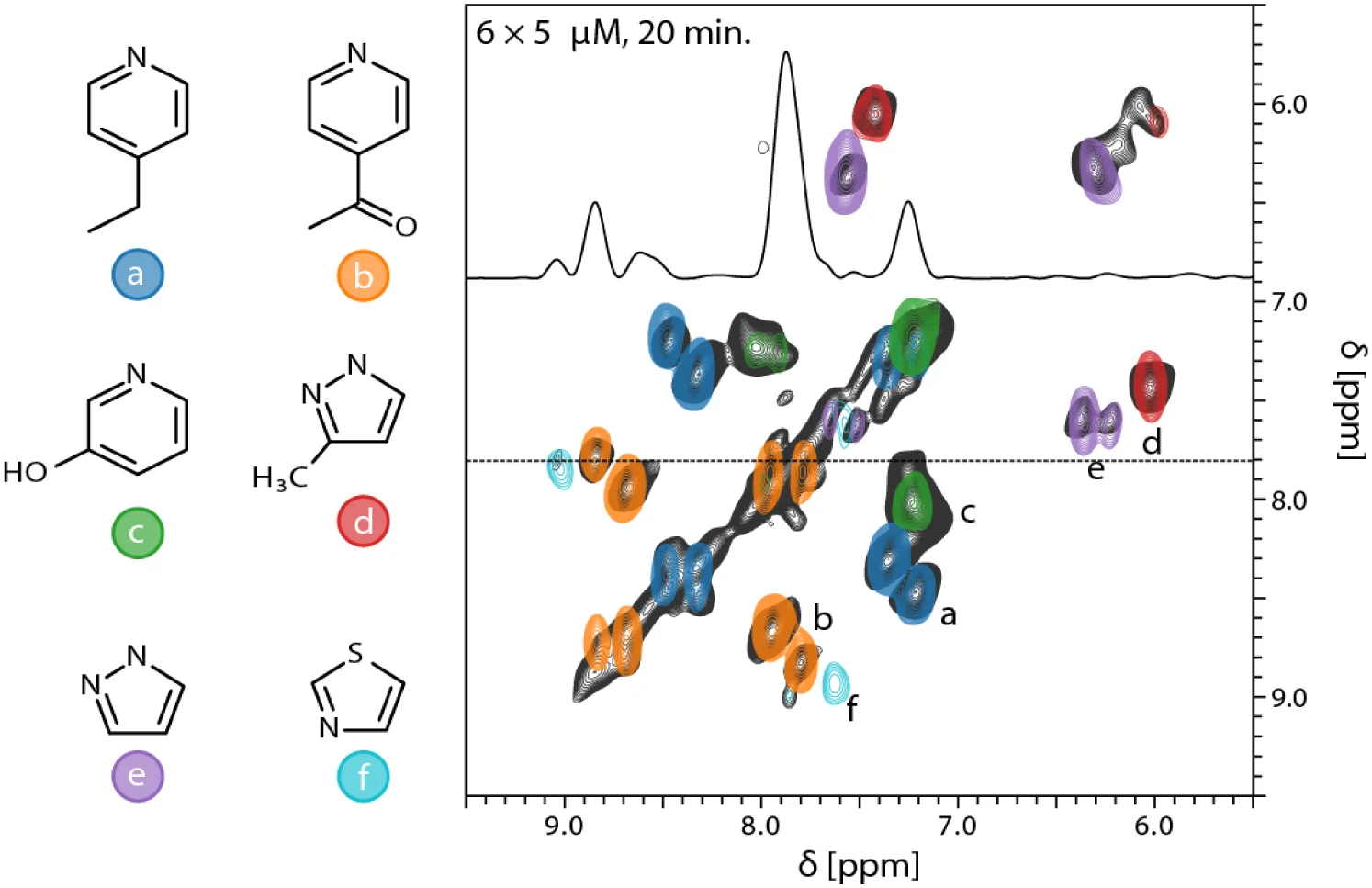
2D SABRE TOCSY spectrum (black) and individual overlays of a mixture of 4-ethylpyridine (blue, a), 4-acetylpyridine (orange, b), 3-hydroxypyridine (green, c), 3-methylpyrazole (red, d), pyrazole (purple, e) thiazole (cyan, f), each at 5 μM, measured at 1 T (43 MHz ^1^H resonance frequency) in 20 min using 100 μM catalyst precursor and 250 μM l-proline as coligand with 10 mM piperidine buffer (pH 11.07) in the presence of 51% para-enriched hydrogen. The reference spectra were measured at thermal equilibrium with 10 mM concentrations, and are all scaled to identical amplitude.

Figure 4 also displays overlays of each component of the mixture measured at thermal equilibrium with 2000× higher concentration and plotted at equal amplitude. Notably, the SABRE spectrum displays a good correspondence with the spectra at thermal equilibrium, with all connectivities being present and well-resolved. This mixture is the same as used for Figure 2; the gain in resolution and structural information is evident.

Although the mixture contains five- and six-membered N-heteroaromatic analytes, substituted at different positions, their 2D TOCSY patterns are sometimes remarkably similar, often resembling those of a two-spin system. This is due to the limited chemical shift dispersion at benchtop field, which can severely hamper structural elucidation and highlights the need for spectral databases for analyte identification. Note that SABRE enhancement within each analyte is not uniform over all protons, and, consequently, the intensity distribution in the hyperpolarized spectrum does not coincide with the reference spectra recorded at thermal equilibrium. It should also be mentioned that the weakness of *J*-coupling interactions can represent a limiting factor for the proposed approach. This is evidenced by the weak correlations observed for thiazole, both in the SABRE and in the reference spectrum, consistent with *J*-coupling values of 2.0 and 3.2 Hz. Despite these limitations, our results indicate a quick route to analyte identification at low micromolar concentrations using benchtop NMR.

The 2D SABRE TOCSY experiment was further tested on a natural extract, namely a methanolic extract of ground coffee. Roasted coffee contains several N-heterocyclic compounds amenable to SABRE hyperpolarization.^30,53,54^ Using SABRE can be particularly advantageous in complex mixtures, as only a subset of compounds is hyperpolarized, effectively providing a chemoselective analysis method. Pyridine- and pyrazine derivatives were previously quantitatively determined at concentrations between 10 and 100 μM in coffee extract using non-hydrogenative ParaHydrogen Induced Polarization (nhPHIP).^55^ This concentration range is compatible with the sensitivity requirements of the 2D SABRE-TOCSY experiment, making a coffee extract a suitable system to test this approach on a complex mixture.

Note that, in contrast to the previously reported nhPHIP approach, SABRE results in signal enhancement of the free analytes, which enables the use of existing spectral databases for analyte identification.

The hyperpolarized 2D TOCSY spectrum of coffee extract, acquired in approximately 1 h, is shown in Figure 5, as black contours. As expected, the spectrum displays a multitude of aromatic, as well as aliphatic signals at ∼2.25 ppm. Based on chemical shifts and information from available literature, several analytes could be identified in the 2D TOCSY spectrum and were thereafter confirmed by measuring pure standards at thermal equilibrium, shown as colored overlays in Figure 5. The region between 7 and 8 ppm is dominated by large signals from 3-hydroxypyridine (green, c), which is known to be present at a high concentration compared to other heteroaromatics.^55,56^ Both pyridine (purple, e) and nicotinic acid (red, d) display complex second-order lineshapes, but their signals are sufficiently resolved to give compelling evidence as to their identity. By virtue of their coupling between aromatic and aliphatic protons, 2,6-dimethylpyrazine (blue, a) and 2-methylpyrazine (orange, b) were identified as well. Cross-peaks resulting from aromatic to aliphatic transfer – occurring in the lower right of the spectrum (∼2.25 ppm, horizontal axis) in Figure 5 – display increased amplitude with respect to their counterparts in the upper left part of the spectrum (∼8.25 ppm in horizontal axis). This is due to SABRE hyperpolarization being higher for the aromatic protons than for the aliphatic protons, which results in several more cross peaks being present in the lower right than in the upper left of the spectrum.

**Figure 5:**
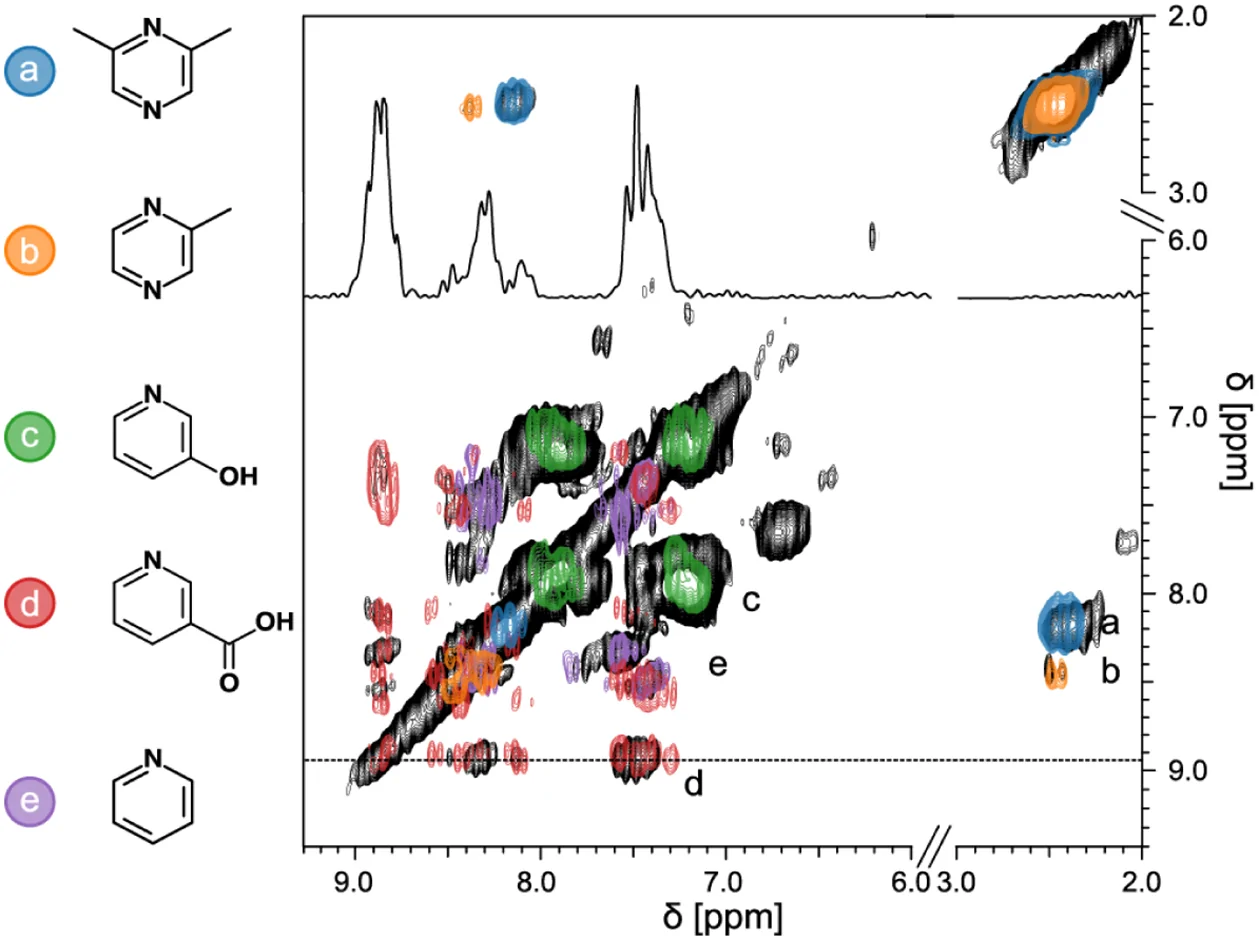
2D SABRE TOCSY spectrum of a mixture of 600 μL methanolic coffee measured at 1 T (43 MHz ^1^H resonance frequency) in 1 h using 450 μM catalyst precursor and 1125 μM l-proline as coligand as well as 10 mM piperidine buffer (pH 11.07) in the presence of 51% *para*-enriched hydrogen. Spectra of identified analytes (2,6-dimethylpyrazine (blue, a), 2-methylpyrazine (orange, b), 3-hydroxypyridine (green, c), nicotinic acid (red, d), pyridine (purple, e)) are overlaid in various colors and further identified with letters in the spectrum.

This analysis of coffee extract represents the first application of 2D SABRE-NMR to elucidate analytes in a complex mixture. While sensitivity is properly addressed by SABRE hyperpolarization, further enhancements of resolving power will be necessary to expand the scope of 2D NMR at benchtop field. Given the strong coupling regime typically pertaining for aromatic compounds at low field, previously proposed pure-shift techniques^57^ are unlikely to provide a viable solution for SABRE-benchtop NMR. Alternatively, heteronuclear SABRE-2D NMR experiments (such as (^13^C, ^1^H) HSQC) would offer significantly increased resolution albeit at the price of ∼100-fold reduction in sensitivity.

## Conclusions

The combination of 2D NMR spectroscopy and SABRE hyperpolarization provides enhanced sensitivity and resolving power, potentially expanding the scope of benchtop spectrometers toward mixture analysis. Our results demonstrate that a 2D SABRE TOCSY experiment, recorded on a mixture of aromatic compounds at low micromolar concentration, can fully resolve a mixture of moderate complexity. The same approach was used to analyze a methanol extract of ground coffee using benchtop NMR and allowed the identification of several aromatic components. Although 2D TOCSY provides increased resolving power compared to 1D spectroscopy, the use of a spectral database remains essential for analyte identification at benchtop field.

Higher sensitivity was obtained compared to previous SABRE approaches, by employing an excess of an α-amino acid as coligand, which resulted in high signal-to-noise for low micromolar concentrations for a 2D SABRE TOCSY, using 51% enriched *para*hydrogen. An additional factor of 3 in enhancement could be gained by using fully enriched *para*hydrogen, leading to a limit of detection at nanomolar levels.

## Supplementary Material



## Notes

Experimental details and supplementary experimental results (PDF)

## Abbreviations

PEP: preservation of equivalent pathways
PHIP: parahydrogen-induced polarization
SABRE: signal amplification by reversible exchange
TOCSY: total correlation spectroscopy
TPPI: time-proportional phase incrementation
LAC: avoided level crossing or level anticrossing
S/N: signal-to-noise
