# Supplementary material for: Mixture Analysis at Micromolar Concentrations with 2D Benchtop NMR Enhanced by SABRE Hyperpolarization
Source: Anal Chem. 2026 Aug 13;98(33):24340–6. doi: 10.1021/acs.analchem.6c02720 (PMC13523550; doi:10.1021/acs.analchem.6c02720)
Supplement: Supplementary file 1 [file ac6c02720_si_001.pdf]

SUPPORTING INFORMATION

Mixture analysis at micromolar concentrations with 2D benchtop-NMR enhanced by SABRE hyperpolarisation

Bono O. Jimmink, Mattia Negroni, Thom B. Posthumus, Arno P.M. Kentgens, Marco Tessari*

Institute for Molecules and Materials (IMM), Radboud University, Heyendaalseweg 135, 6525 AJ Nijmegen, The Netherlands.

TABLE OF CONTENTS

Chemicals	S-2
Coffee extraction procedure	S-2
Comparison of L-proline and glycine as co-ligands for SABRE	S-3
Full 2D TOCSY spectrum of SABRE enhanced coffee extract	S-3
References	S-4

Chemicals

Methanol was purchased from Fisher Scientific. Methanol-d_4, 3-hydroxypyridine, 4-acetylpyridine, thiazole, 4-ethylpyridine, 4-methylpyrazole, pyrazole, DMSO, L-proline, pyrazine, piperidine, piperidine.HCl, 2,6-dimethylpyrazine, 2-methylpyrazine and nicotinic acid were purchased from Sigma Aldrich. Pyridine-d_5 was purchased from deuteron GmbH. Pyridine was purchased from Fluka chemicals. All chemicals were 95% purity or higher and were used without further purification. The pre-catalyst complex [Ir(SIMes)(COD)]Cl was synthesised according to the procedure described by Kelly III et al.[1] A parahydrogen generator (Hyperspin scientific) cooled by liquid nitrogen to 77 K was used to convert thermal hydrogen (purity 5.0, Linde Gas Benelux B.V.) to 51% para-enriched hydrogen. Nitrogen gas was obtained from the on-site bulk nitrogen supply (Linde).

Coffee extraction procedure

The procedure for preparing the methanolic extract of ground coffee was adapted from Hermkens et al.[2] In short: Vacuum packed finely ground coffee was obtained from a local supermarket in The Netherlands. The coffee was declared as a dark roast of arabica and robusta beans. The extraction was performed by suspending 1.8 g of ground coffee in 5 ml CD_3OD and vigorously stirring this suspension at room temperature for 30 minutes. Thereafter, the solution was centrifuged by a hand-centrifuge and filtered through a 0.22 μM syringe filter. A 600 μl aliquot of this extract was used to prepare a sample with a total volume of 1500 μl, meaning a dilution factor of 2.5x.

Comparison of L-proline and glycine as co-ligands for SABRE

Two samples were prepared with the composition: 300 μM [Ir(SIMes)(COD)]Cl, 600 μM co-ligand, 5 mM piperidine buffer, 300 μM 3-hydroxypyridine and 300 μM 2-methylpyrazine in CD_3OD, spiked to 0.5% CH_3OH. Where the co-ligand is either L-Proline or Glycine. The spectra shown below indicate that both co-ligands work to a similar extend.

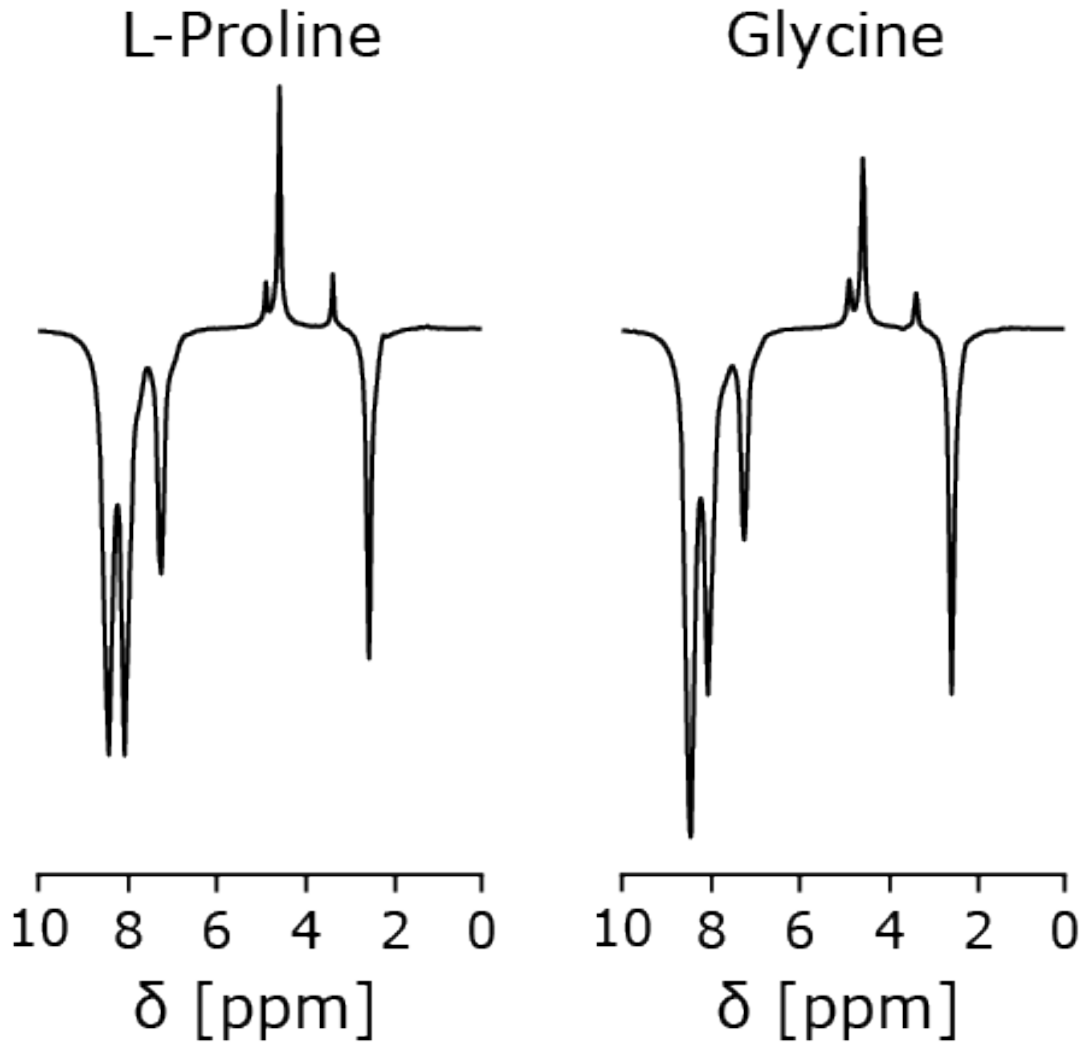

Figure S1. Comparison of SABRE spectra recorded for 2-methylpyrazine and 3-hydroxypyridine using L-Proline (left) or Glycine (right) as co-ligand.

Full spectrum of hyperpolarised coffee extract

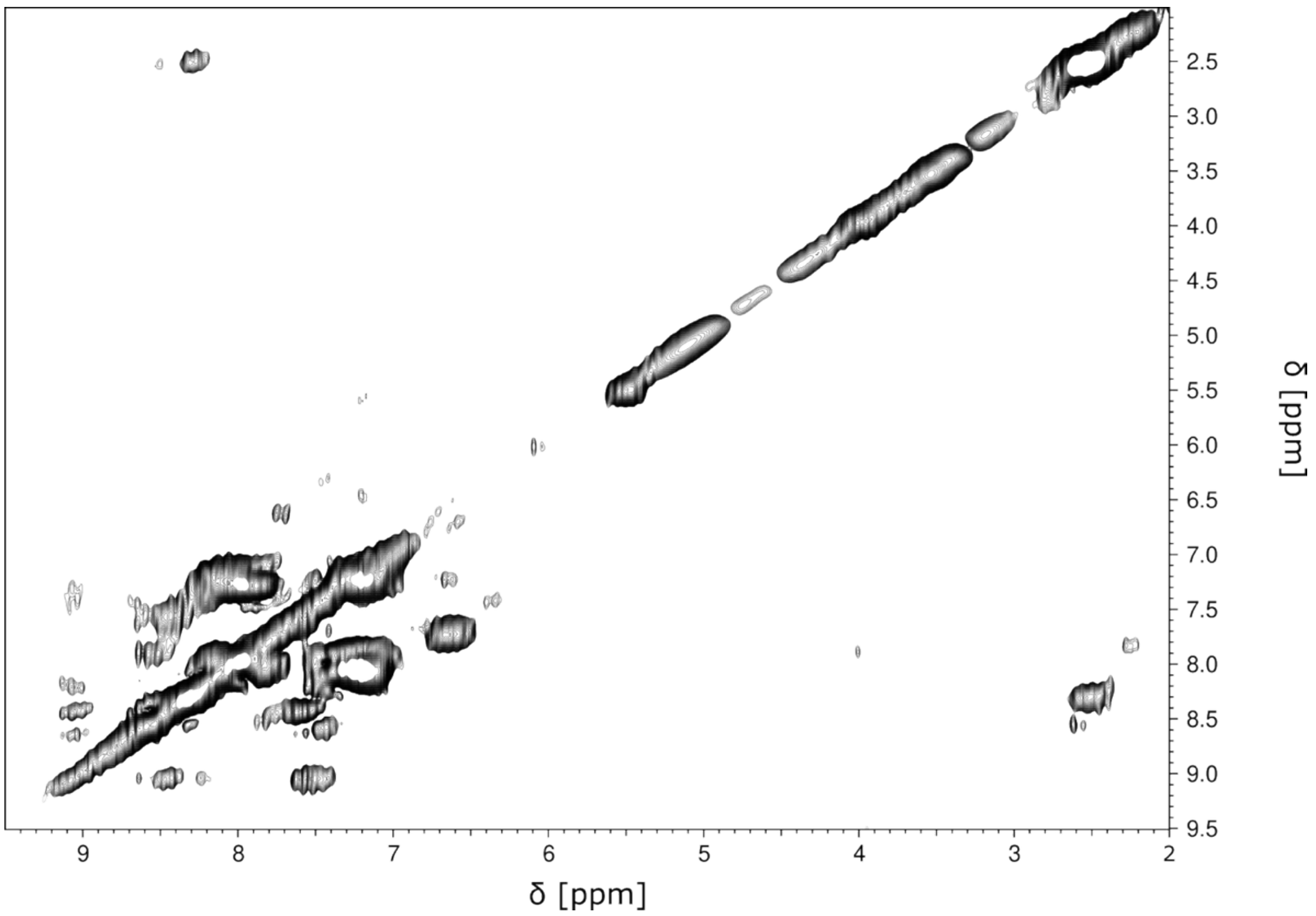

Figure S2. 2D SABRE TOCSY spectrum of a mixture of 600 μl methanolic coffee extract measured at 1 T (43 MHz ^{1}H resonance frequency) in 1 hour using 450 μM catalyst precursor and 1125 μM L-proline as co-ligand as well as 10 mM piperidine buffer (pH 11.07) in the presence of 51% *para*-enriched hydrogen.

References

(1) Kelly III, R.A.; Clavier, H.; Giudice, S.; Scott, N.M.; Stevens, E.D.; Bordner, J.; Samardjiev, I.; Hoff, C.D.; Cavallo, L.; Nolan, S.P., Determination of N-heterocyclic carbene (NHC) steric and electronic parameters using the [(NHC)Ir(CO)2Cl] system. *Organometallics* **2008**, *27* (2), 202-210.

(2) Hermkens, N. K. J.; Eshuis, N.; Van Weerdenburg, B. J. A.; Feiters, M. C.; Rutjes, F. P. J. T.; Wijmenga, S. S.; Tessari, M. NMR-Based Chemosensing via *p* -H_2 Hyperpolarization: Application to Natural Extracts. *Anal. Chem.* **2016**, *88*
